# Associations between symptoms of eating disturbance and frequency of physical activity in a non-clinical, population-based sample of adolescents

**DOI:** 10.1186/s40337-019-0239-1

**Published:** 2019-04-18

**Authors:** Ove Heradstveit, Eva Holmelid, Helene Klundby, Birgitte Søreide, Børge Sivertsen, Liv Sand

**Affiliations:** 1 Regional Centre for Child and Youth Mental Health and Child Welfare, Ove Heradstveit, NORCE Norwegian Research Centre, RKBU, Nygårdsgaten 112, 5008 Bergen, Norway; 2 Center for Alcohol & Drug Research, Stavanger University Hospital, Stavanger, Norway; 3 Faculty of Psychology, University of Bergen, Bergen, Norway; 4 Department of Health Promotion, Norwegian Institute of Public Health, Bergen, Norway; 5 Department of Research & Innovation, Helse Fonna HF, Haugesund, Norway; 6 Department of Mental Health, Norwegian University of Science and Technology, Trondheim, Norway

**Keywords:** Eating disturbance, Disordered eating, EDS-5, Physical activity, Population-based study, Sex differences, Correlates, Heterogeneity

## Abstract

**Background:**

Physical activity is an important factor related to eating disorders, but the relationship between symptoms of eating disorders and physical activity is multifaceted. The aims of this study were to investigate how symptoms of eating disturbance (ED) were associated with physical activity, and to explore potential sex differences and the potential moderating effects from body mass index (BMI) scores.

**Methods:**

Data stem from a large population-based survey of 10,172 Norwegian adolescents aged 16 to 19 years, the youth@hordaland-survey. The main dependent variable was self-reported number of days with physical activity per week, while the main independent variable was self-reported symptoms of ED using the five-item Eating Disturbance Screening (EDS-5) questionnaire. Control variables included sex, age, socioeconomic status, and BMI.

**Results:**

Girls reported substantially more symptoms of ED compared with boys (*M* = 3.02 versus 1.32, *d* = 0.80, *p* < 0.001), as well as fewer days with physical activity per week (*M* = 2.88 versus 3.46, *d* = − 0.28, *p* < 0.001). For both sexes, symptoms of ED were negatively associated with physical activity (adjusted mean differences (adj. mean diff) ranging from − 0.03 to − 0.08, all *p* < 0.05). Interaction analyses showed, however, that associations between symptoms of ED and physical activity were significantly moderated by BMI scores for both girls (*p* < 0.01) and boys (*p* < 0.05). Specifically, ED symptoms were associated with lower physical activity levels among adolescents with higher BMI scores.

**Conclusions:**

The present study indicates that symptoms of ED were overall negatively associated with physical activity for both sexes during adolescence. However, associations between ED symptoms and physical activity levels differed considerably across the weight spectrum.

## Plain english summary

Physical activity is related to eating disorders in multifaceted ways. This study aimed to disentangle how symptoms of eating disturbance (ED) relate to physical activity in a non-clinical, community-based sample. The study also investigated how these associations varied across sexes and across the weight-spectrum. A cross-sectional design was used, employing data from a large, Norwegian population-based study, the youth@hordaland-survey. The study included 10,172 adolescents that all reported symptoms of ED as well as physical activity levels per week. In addition, age, socioeconomic status, and body mass index were included in the analyses as covariates. The study revealed that symptoms of ED were negatively associated with physical activity for both sexes. However, body mass index (BMI) moderated these associations, revealing that ED symptoms were associated with lower physical activity levels specifically among adolescents with higher BMI scores.

## Introduction

Although eating disorders are rare in the general population, they are relatively more common among adolescent girls and young women compared with boys and young men [1, 2]. In a large, representative sample of US adolescents aged 13 to 18 years, the lifetime prevalence of anorexia nervosa (AN), bulimia nervosa (BN), and binge-eating disorders (BED) were estimated to be 0.3, 0.9 and 1.6%, respectively [3], with higher prevalence in girls compared with boys [3]. Hence, BED is the most common eating disorder, both in adolescence [3] and adulthood [4]. Importantly, there is a considerable heterogeneity in the symptom expression across different subtypes of eating disorders. Individuals with AN are underweight [5, 6]; individuals with BN have higher body mass index (BMI) than those without any eating disorder [6]; and over 65% of individuals with BED have a BMI > 30 [7].

The link between eating disturbances (ED) and physical activity is well documented (e.g. [8, 9]), and several studies have demonstrated associations between symptoms of eating disorders and excessive exercise (for a review, see [10]). Particularly, high levels of exercise are linked with both AN and BN [11]. One study found that excessive physical activity was highest among those with purging type AN [11], while another study reported the highest levels of compulsive exercise in AN restrictive type [12]. However, weight-related differences may have relevance for physical activity levels, as several studies have demonstrated negative associations between BMI and physical activity [13–15]. BED in females was in one study found to be associated with lower physical activity levels compared with counterparts without the disorder [16]. Another study found that high BMI individuals with BED had significantly lower physical activity levels compared with BMI-matched individuals without BED [17]. On the other hand, a recent study by Barber and colleagues [14] with overweight adults showed low levels of physical activity in this group, while BED symptoms were unrelated to physical activity amongst the overweight individuals. Thus, the literature is inconclusive to whether or not symptoms of eating disorders are independently related to physical activity levels when also weight/BMI is taken into account.

Most previous studies on associations between eating disorders and physical activity have been occupied with excessive or compulsive physical activity. Less attention has been given to how symptoms of eating disorders may also be linked with frequency of physical activity. Moreover, a large proportion of previous studies have used data from clinical samples, in which female patients are overrepresented, and in which eating disorders are often restricted to only include individuals suffering with AN/BN [10]. Only a minority of people who meet stringent diagnostic criteria for eating disorders are, however, seen in mental health care [2], and there is a need to supplement studies based on clinical population with data from more general populations. Although few in number, there are some studies that have explored associations between symptoms of ED and frequency of physical activity employing non-clinical, population-based samples of adolescents. Hay and colleagues [6] found no significant differences in amount of physical activity in adolescents with symptoms of eating disorders compared with the general adolescent population. An interesting, but small study from Gomes and colleagues [18] found that adolescents who exercised regularly showed fewer symptoms of eating disordered behaviors.

The existing scientific knowledge base is also rather scarce in regards to whether associations between symptoms of ED and physical activity differ across sexes, something that was investigated in the present study. Previous publications show that girls more frequently exhibit symptoms of eating disorders [3], and also display lower levels of overall physical activity than boys [19]. In addition, expression of eating disorders may vary across sexes [20, 21], to the extent that it has been suggested that eating disorders differ structurally among boys and girls [21–24]. All these findings highlight the possibility that symptoms of ED may be differentially associated with physical activity among respectively boys and girls.

In the present study, we investigated how symptoms of ED – using the five-item Eating Disturbance Scale (EDS-5) [25] – were associated with frequency of physical activity in a general adolescent population, and to what extent these associations varied across sexes. Importantly, we also analyzed the potential confounding effects from sociodemographic variables and BMI, as well as the potential moderating effects of BMI on the associations between symptoms of ED and physical activity. Based on the previous findings from Gomes and colleagues [18] we hypothesized that total symptoms of ED would be overall negatively associated with frequency of physical activity. However, we expected to find moderating effects from BMI on these associations, as BED has been linked with overweight and lower physical activity [17], while AN has been linked with underweight and excessive exercise [11, 12].

## Method

### Participants

This study used data from the youth@hordaland survey. This is a total population study aimed at all adolescents in the county of Hordaland born between 1993 and 1995. The aim of the youth@hordaland survey was to obtain information to improve children and adolescents’ mental health care, as well as knowledge about life style, school function and family situation. The present study includes data collected in 2012 from the youth@hordaland survey. Ten thousand two hundred fifty-seven adolescents aged 16–18 years answered the questionnaire. As previous studies frequently apply 15 as a lower and 50 as the upper limit of BMI ranges [26], we excluded participants with outlier scores on BMI (< 15 or > 50, *N* = 85), and the final sample therefore comprised 10,172 individuals.

### Materials

#### Eating disturbance (ED) symptoms

In the youth@hordaland survey, the Eating Disorders Scale (EDS-5) was used as a measure of symptoms of ED. The EDS-5 is based on self-report and consists of five questions considering eating, developed by Rosenvinge and colleagues [25]. Although the instrument has not been specifically validated in adolescents, it has shown adequate discriminative validity in detecting symptoms of eating disorders in students (mean age 25.6 years) [25]. Particularly, the EDS-5 score demonstrated high correlations with concern about weight (R = 0.89) and concern about shape (R = 0.85), and also adequate correlations with concern about eating (R = 0.70), dietary restraint (R = 0.61) and overeating/bulimia (R = 0.60) [25].

The EDS-5 items address discontent with own eating habits (item 1), comfort eating (item 2), feelings of guilt related to eating (item 3), strict dieting in order to gain control over eating (item 4), and thoughts of being too fat (item 5). The answers of each item were rated on a Likert scale from 0 (“not true”), 1 (“sometimes true”) and 2 (“certainly true”). In order to calculate a total score, we reversed the first item of the EDS-5 questionnaire (“I am satisfied with my eating habits”), which was the only variable in which the response “not true” represented an indication of ED. Other items were indicative of a problem score when the response was “certainly true”, for example “I have feelings of guilt related to eating.” We then summed the items of each EDS-5 item into a continuous variable for total EDS-5 score, which indicated total levels of ED. The summed variable was used in our analyses as a continuous measure of ED ranging from 0 to 10 (*M* = 2.23, *SD* = 0.23).

The EDS-5 instrument has previously been shown to have a high sensitivity (.90) and specificity (.88) to the fourth edition of the Diagnostic and Statistical Manual for Mental Disorders (DSM-IV) criteria for eating disorders in the general young adult population, while internal reliability analyses showed a Cronbach’s alpha of 0.83 [25].

We conducted a principal component analysis on the EDS-5 questionnaire in our sample. This analysis was conducted in order to indicate if symptoms of ED were best represented as a one-factor construct, as well as to evaluate the factor loading of each item. The results from this analysis informed the further operationalization of ED symptoms in this study. We found only one factor with an eigenvalue of 1 or higher, accounting for a total of 52% of the variance for girls and 46% of the variance for boys (Table 1). The factor loadings were overall strong for each of the five items (ranging from 0.60 to 0.84 for girls, and from 0.71 to 0.82 for boys). The only exception was item 1 (discontent with own eating habits) that had a considerably lower factor loading for boys (0.35), and below the threshold of .50 indicated by Hair et al. [27] of being acceptable. In our population, the EDS-5 demonstrated acceptable reliability for girls, measured by an overall Cronbach’s Alpha of 0.76. For boys the reliability was somewhat lower for the five-item EDS-5 scale (α = 0.64). Therefore, we also constructed a revised EDS total score for boys, which only EDS-items 2, 3, 4 and 5 (item 1 was omitted). This four-item scale performed acceptable for boys (α = 0.71), and was used for boys in the secondary analyses.

**Table 1:** Principal component analysis of the five items in the EDS-5 questionnaire

	Factor loadings on 1 component	
	Girls (*n* = 5266)	Boys (*n* = 4611)
EDS Item 1	0.68	0.35
EDS Item 2	0.60	0.71
EDS Item 3	0.84	0.82
EDS Item 4	0.67	0.71
EDS Item 5	0.79	0.71
Total variance accounted for by component	51.8	46.0

Eigenvalue for 1 factor: 2.59 for girls, 2.30 for boys

#### Physical activity

The youth@hordaland survey included a question to investigate levels of physical activity. Specifically, the adolescents were asked to respond to how many of the past 7 days they engaged in physical activity for at least 60 min. The answers on this item ranged from 0 to 7. A continuous variable spanning from 0 to 7 was constructed (*M* = 3.15, *SD* = 0.02), and was used in all our analyses.

#### Body mass index (BMI)

Data on height in cm and weight in kg were collected by self-report, and body mass index (BMI) was calculated by dividing the adolescent’s weight in kilograms by his/her height in meters squared. We also standardized BMI scores separately for each sex.

#### Sociodemographic variables

We collected information on sex and age of the participants from the National Population registry. Socioeconomic status (SES) was measured in the youth@hordaland survey by three separate variables: mother’s education, father’s education and perceived family financial circumstances. Responses on both the variables of mother’s and father’s education which were categorized as “primary school”, “secondary school” and “college or university” Perceived family financial circumstances (i.e. how well off the adolescent perceived their family to be) were assessed by asking the adolescents about their family financial circumstances compared with most others. The response options were [1] “better financial circumstances”, [2] “approximately like most others” and [3] “poorer financial circumstances”. The three SES variables were included separately in the regression analyses.

### Procedure

The youth@hordaland study is a cross-sectional population-based study carried out during early 2012, and data was collected from adolescents in upper secondary school. The adolescents received information per email and one school hour was used to complete the questionnaires at school. In addition, adolescents not going to school received the questionnaires by mail at their home address, and mental health services and other institutions were contacted to let adolescents from these settings participate. The questionnaires used in the youth@hordaland study were web-based, and electronic informed consent was obtained from all participants. The Regional Committee for Medical Research in Western Norway approved the study.

### Design and analyses

First, we conducted descriptive analyses of the sample. We used t-tests for independent samples and Pearson chi-square tests to indicate sex differences in the participants’ age, SES, BMI, weekly physical activity levels, and symptoms of ED. In addition, Cohens’ d effect size of mean differences across sex were reported where applicable. Second, mean BMI levels were calculated for individuals with problem scores on each of the EDS-items, stratified by sex. Third, linear regression models were conducted for associations between continuous levels of symptoms of ED (i.e. EDS-5 total scores) and mean number of days with physical activity per week, stratified by sex. Specifically, we analyzed the associations between ED symptoms and physical activity in an unadjusted model, and after adjustment for sociodemographic variables and BMI. Finally, we analyzed interactions between ED symptoms and BMI scores for each sex in the linear prediction of physical activity. The results were visualized with margins plot of the predicted number of days with physical activity per week across increasing symptoms of ED stratified by high and low BMI scores. Specifically, symptoms of ED spanned from 0 to 10 on the EDS-5 scale. We used standardized BMI scores, in which low BMI scores were defined as two standard deviations below sex-specific mean and high BMI scores were defined as two standard deviations above the sex-specific mean. All data were analyzed using Stata version 14.

## Results

**Fig. 1:**
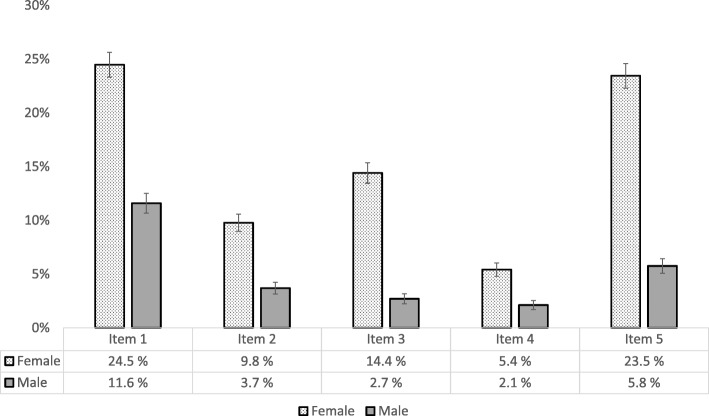
Distribution of positive scores on single symptoms of eating disturbance stratified by sex (*N* = 10,172)^1^. ^1^ The error bars indicate the 95% confidence interval of each estimate. Includes items 1–5 on the EDS-5 questionaire

As outlined in Table 2, the sample comprised significantly more girls (52.8%), and girls were slightly older compared with boys (17.45 versus 17.41 years, *d* = 0.04, *p* < 0.05). Girls reported somewhat lower levels of perceived family economic resources (*p* < 0.001) and had significantly lower mean BMI compared with boys (22.58 versus 21.96, *d* = − 0.17, p < 0.001). Girls also reported lower mean number of days with physical activity per week (3.46 versus 2.88, *d* = − 0.28, *p* < 0.001), and had considerably higher mean levels on symptoms of ED compared with boys (3.02 versus 1.32, *d* = 0.80, *p* < 0.001). Figure 1 outlines the distribution of positive scores on each individual EDS-5 item stratified by sex.

**Table 2:** Descriptive characteristics of the sample (*N* = 10,172)

	Girls (*n* = 5371)	Boys (*n* = 4801)	Cohens d	*p*-value
Sociodemographic variables and BMI				
Sex, *N* (%)	5371 (52.8)	4801 (47.2)	–	<.001
Age, *M* (*SD*)	17.45 (0.85)	17.41 (0.84)	.04	.039
Perceived family economic resources, *N* (%)			–	<.011
- Poorer financial circumstances	413 (7.9)	289 (6.2)		
- Approximately like most others	3670 (70.1)	2968 (64.0)		
- Better financial circumstances	1150 (22.0)	1378 (29.7)		
Mother’s education, *N* (%)^a^			–	.157
- Primary school	1664 (40.3)	1470 (42.4)		
- Secondary school	447 (10.8)	336 (9.7)		
- University / college	2021 (48.9)	1663 (47.9)		
Father’s education, *N* (%)^b^			–	.272
- Primary school	1786 (45.8)	1670 (47.3)		
- Secondary school	425 (10.9)	367 (10.4)		
- University / college	1688 (43.3)	1496 (42.3)		
Body mass index, *M* (*SD*)	21.96 (3.58)	22.58 (3.56)	−.17	<.001
Physical activity				
Mean number of days per week, *M* (*SD*)	2.88 (0.03)	3.46 (0.03)	−.28	<.001
Eating disturbance				
EDS-5 total score, *M* (*SD*)	3.02 (2.49)	1.32 (1.64)	.80	<.001

^a^Includes only 7.601 individuals, due to *n* = 2571 having answered that they don’t know

^b^Includes only 7.432 individuals, due to *n* = 2740 having answered that they don’t know

As highlighted in Table 3, individuals that reported symptoms of ED had higher mean levels of BMI compared with individuals with non-problem scores in the adolescent sample. This pattern was identified for each of the included EDS-5 items across sex.

**Table 3:** Mean body mass index levels across problem scores on symptoms of eating disturbance in the sample (*N* = 10,172)

	Girls (*n* = 5371)	Boys (*n* = 4801)
	M (SD)	M (SD)
Full sample	22.0 (0.05)	22.6 (0.05)
Item 1: Discontent with own eating habits^a^	22.7 (0.12)^b^	23.4 (0.20)^b^
Item 2: Comfort eating^a^	23.2 (0.20)^b^	24.1 (0.39)^b^
Item 3: Feelings of guilt related to eating^a^	23.1 (0.16)^b^	24.7 (0.48)^b^
Item 4: Strict dieting to gain control over eating^a^	23.1 (0.28)^b^	24.2 (0.53)^b^
Item 5: Thoughts of being too fat^a^	24.2 (0.13)^b^	27.2 (0.30)^b^

^a^Include individuals with problem scores on the indicated EDS-5 item

^b^Problem scores on each of the EDS-5 items were significantly associated (*p* < 0.001) with higher mean BMI compared with non-problem scores

Table 4 outlines results from linear regression analyses of associations between symptoms of ED and number of days with physical activity per week. Symptoms of ED were negatively associated with physical activity in unadjusted models for both girls (mean diff. = − 0.04, *p* < 0.001) and boys (mean diff. = − 0.09, *p* < 0.001), and in the adjusted model accounting for the potential confounding effects from age, SES and BMI, for both girls (adj. mean diff = − 0.03, *p* < 0.05) and boys (adj. mean diff = − 0.08, p < 0.05). Secondary analyses were conducted for boys with the ED scale that omitted item 1, and the results were very similar to the results using the full EDS-5 scale (not shown).

**Table 4:** Associations between total symptoms of eating disturbance and levels of physical activity (*N* = 10,172)^a^

	Girls (*n*=5,371)		Boys (*n*=4,801)^a^	
	Mean diff / adj. mean diff (95% CI)	*p*-value	Mean diff / adj. mean diff (95% CI)	*p*-value
Unadjusted model				
- Symptoms of eating disturbance (ED)	-0.04 (-0.07, -0.02)	<.001	-0.09 (-0.13, -0.04)	<.001
Adjusted model				
- Symptoms of eating disturbance (ED)	-0.03 (-0.05, -0.01)	.049	-0.08 (-0.14, -0.02)	.013
- (+) adjusted for BMI^b^	-0.01 (-0.03, 0.01)	.168	-0.02 (-0.04, 0.01)	.197

^a^ Excluding item 1 from total EDS-5 score due to low factor loading of item on eating disturbance concept

^b^ The adjustment for BMI follows the additional adjustment for age and socioeconomic status (not shown)

**Fig. 2:**
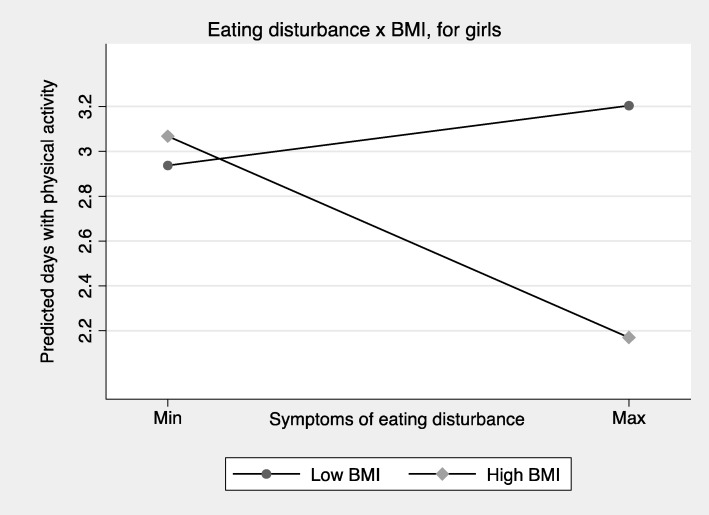
Number of days with physical activity predicted from symptoms of eating disturbance across BMI scores, for girls (*n* = 5371)^1^. ^1^ Presents the predicted mean number of days with physical activity from symptoms of eating disturbance, stratified by high versus low BMI scores. Low BMI is defined here as two standard deviations below the sex-specific mean BMI, while high BMI is defined by two standard deviations above the mean BMI

**Fig. 3:**
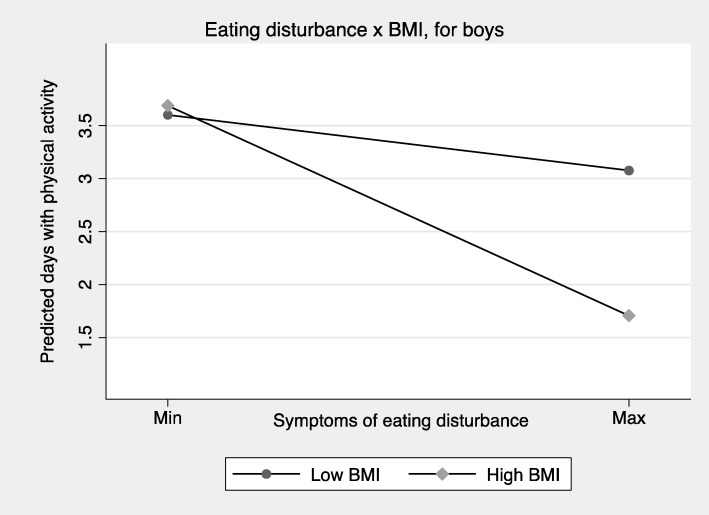
Number of days with physical activity predicted from symptoms of eating disturbance (EDS-5, full scale) across BMI scores, for boys (*n* = 4801)^1^. ^1^ Presents the predicted mean number of days with physical activity from symptoms of eating disturbance, stratified by high versus low BMI scores. Low BMI is defined here as two standard deviations below the sex-specific mean BMI, while high BMI is defined by two standard deviations above the mean BMI

Moderation analyses showed that the interaction between total symptoms of ED and BMI was significant for both girls (*p* < 0.01) and boys (p < 0.05). Figures 2 and 3 illustrate that symptoms of ED were negatively associated with physical activity at the higher end of BMI scores. In addition, ED symptoms among girls in the lower end of BMI showed a tendency of a positive association with physical activity. In secondary analyses, we used the four-item ED symptoms scale for boys, and the interaction between symptoms of ED and physical activity remained significant (*p* < 0.01) (figure not shown).

## Discussion

The present study demonstrates that despite considerable sex differences in levels of physical activity and symptoms of ED in a general sample of adolescents, associations between symptoms of ED and physical activity were overall similar for boys and girls. For both sexes, symptoms of ED were negatively associated with physical activity. However, these associations were significantly moderated by BMI. Specifically, for adolescents with higher BMI, symptoms of ED were negatively associated with physical activity, while this pattern was not observed for adolescents with lower BMI. On the contrary, symptoms of ED tended to be positively associated with physical activity for girls with lower BMI.

### Eating disturbance and physical activity

The findings from the present study underscore the multifaceted relationship between disturbed eating symptoms and physical activity. We found an overall *negative* association between symptoms of ED and physical activity, corresponding with a similar study by Gomes and colleagues [18]. They reported lower levels of ED symptoms in adolescents that were regularly physical active compared with those with lower physical activity. On the other hand, our results provide less support to findings from Hay and colleagues [6] that reported no significant differences in amount of physical activity in adolescents with symptoms of eating disorders compared with the general adolescent population. As BED is the most prevalent eating disorder among adolescents [3], and is related to overweight as well as lower physical activity levels [17], it is possible that BED may account for this negative association entirely. However, our lack of data on specific eating disorders in the sample makes this assumption difficult to validate.

The present study demonstrates that associations between symptoms of ED and physical activity differ considerably across the weight-spectrum. The interaction analyses demonstrated that negative associations between symptoms of ED and physical activity were seen specifically among individuals with high BMI scores. These findings may be interpreted in the context of the large heterogeneity between different types of eating disorders, and their potentially differential associations with physical activity. Previous studies have demonstrated that dietary constraints are often observed in individuals with AN [28] and BN [29], and that higher levels of eating disorder symptoms [11], drive for thinness [30], and body dissatisfaction [30, 31] are other features among eating disordered individuals that are associated with excessive exercise. On the other hand, lower physical activity levels are previously demonstrated in relation to BED for females [16]. The present study adds to this knowledge base by demonstrating that ED was negatively associated with physical activity across sexes, and that BMI scores were an important moderator in the association between symptoms of ED and frequency of physical activity.

As expected from previous findings [3, 19], a range of sex differences were found in both the distribution of physical activity levels, symptoms of ED, and mean BMI scores. Boys had significantly higher frequency of physical activity compared with girls, lower mean symptoms of ED, and a somewhat higher mean BMI. Hence, the present study confirms the need to target girls selectively in interventions aiming at promoting physical activity in adolescents. On the other hand, both boys and girls with high BMI in combination with symptoms of ED had marked lower frequency of physical activity.

Our findings may suggest that symptoms of ED exacerbate sedentary lifestyle problem characterized by a general lack of physical activity among overweight individuals. These findings are aligned with previous studies that reported lower physical activity among individuals with BED [16, 17]. Individuals with BED typically exhibit excessive concerns with their body shape and lack of thinness and spend more time on dieting efforts than healthy controls [32]. Interestingly, all the distinct symptoms of ED included in the present study were associated with higher BMI. Future studies should therefore also investigate how features of eating disorders that correlate with lower BMI are associated with frequency of physical activity in non-clinical, population-based samples.

### Strengths and limitations

A considerable strength of the present study is the application of a large, population-based sample. These data provided a promising approach to disentangle the multifaceted relationship between symptoms of eating disorders and physical activity in samples that is not biased by selection into mental health services [2]. Sex-specific analyses add as another strength of the study. We included frequency of physical activity as our outcome measure, something that seem important in light of previous literature that demonstrate significant links between symptoms of eating disorders and physical activity in both ends of the physical activity continuum. In addition, we adjusted our analyses for BMI, something that is useful in light of the likely correlation between BMI and physical activity levels [13–15]. Not least, BMI scores were also used in moderation analyses, highlighting the important role of weight characteristics in associations between symptoms of ED and frequency of physical activity.

The present study also has some limitations. First, the EDS-5 questionnaire applied in the present study is highly correlated with DSM-IV-defined eating disorders [25], but do not imply the presence of eating disorder diagnoses. Although the EDS-5 scale has been validated among young adults [25], there is still a need for studies that provide firm support of the suitability of the questionnaire in even younger adolescent populations. A principal component analysis of the five items in the EDS-5 in our sample supported a one-factor model of ED for both sexes, and the reliability of the five-factor scale were acceptable for girls, while the reliability was lower for boys. However, a four-item scale for symptoms of ED in boys (omitting item 1 from the EDS-5), provided an acceptable reliability, and supported the findings from analyses done with the full EDS-5 scale. Future studies should aim to replicate our findings using more rigorous measures of ED. Moreover, all the single symptoms of ED were associated with higher BMI and it is likely that they primarily represent non-restrictive patterns of eating disturbances. Thus, there is a need for future investigations into associations between specific symptoms of ED and physical activity that apply comprehensive measures of symptoms of ED including restrictive psychopathology. Second, the study has a cross-sectional design and we cannot conclude the directionality of the associations between symptoms of ED and physical activity. It is likely that physical activity levels as well as other lifestyle factors are reciprocally associated with symptoms of ED, and longitudinal studies on this subject are needed. Third, we only used one self-reported measure of weekly frequency of physical activity, in which the subjects reported days with physical activity “at least 60 minutes” per day. Some adolescents may exercise regularly but for shorter periods than an hour. In addition, girls may potentially be more reluctant to endorse that they were physically active over this cut-off compared with boys. Furthermore, we did not have any possibility to evaluate the quality of physical activity. More comprehensive physical activity instruments assessing both the frequency, duration and intensity of physical activity would have added strength to our findings. Associations between ED and the quality and character of exercise remain to be studied in large, population-based samples of adolescents.

### Implications

The present study highlight the importance of being aware of reduced levels of physical activity among adolescents with symptoms of ED, specifically in the higher end of the weight-spectrum. The literature has previously highlighted that excessive physical activity is an important correlate of ED, as it can be viewed as a risk- and maintaining factor [31], as well as a symptom of some types of eating disorders [5]. The present study adds to this knowledge base, supporting previous findings that also pointed to low physical activity as a correlate of ED [16, 18].

## Conclusions

The present study underscores the close relationship between symptoms of ED and low physical activity among adolescents, and that this was moderated by BMI.

## Abbreviations

BMI: Body mass index
DSM-IV: Diagnostic and Statistical Manual of Mental Disorders, fourth edition
ED: Eating disturbance
EDS-5: Five-item Eating Disturbance Screening questionnaire
SES: Socioeconomic status
